# Experimental inoculation of chicken broilers with *C. gallinacea* strain 15-56/1

**DOI:** 10.1038/s41598-021-03223-w

**Published:** 2021-12-13

**Authors:** Monika Szymańska-Czerwińska, Agnieszka Jodełko, Kinga Zaręba-Marchewka, Krzysztof Niemczuk

**Affiliations:** 1 Department of Cattle and Sheep Diseases, National Veterinary Research Institute, Puławy, Poland; 2 Laboratory of Serological Diagnosis, National Veterinary Research Institute, Puławy, Poland

**Keywords:** Microbiology, Zoology

## Abstract

*Chlamydia gallinacea* is one of the new *Chlamydia* species, encountered predominantly in birds and occasionally in cattle, and its dissemination, pathogenicity and zoonotic potential have not yet been fully elucidated. Until now, no case of clinical infection has been described in poultry, but the number of studies is limited. This study was conducted to evaluate the course of infection and the impact on production parameters in chicken broilers inoculated with the strain 15-56/1 isolated from a Polish flock. The presence of *C. gallinacea* was confirmed in oropharyngeal and cloacal swabs by real-time PCR from the fifth day post inoculation (dpi). Pathogen DNA was also detected in many internal organs of inoculated chickens. All infected animals remained asymptomatic during the entire experimental period, although statistical analyses showed that broilers in the experimental group exhibited significantly lower body weight gains and feed conversion ratios than animals in the control group. These data indicate that subclinical *C. gallinacea* infection in broilers may lead to financial losses for poultry farmers.

## Introduction

Avian chlamydiosis is an infectious disease of domestic and wild birds caused by different *Chlamydia* species. Until recently, *C. psittaci* has been considered the dominant chlamydia agent in birds, although new avian species have been described ^1,2^. One of these, *Chlamydia gallinacea*, primarily infects poultry, but has also been detected in Chinese cattle ^3^. Its presence has been recorded in chickens, ducks, guinea fowl, turkeys and other domestic poultry in several countries, including Poland ^4–8^. Infections with *C. gallinacea* have also been detected in wild birds: in the Australian galah (*Eolophus roseicapillus*) and crimson rosella (*Platycercus elegans*) in Australia ^8,9^ and the Eurasian woodcock (*Scolopax rusticola*) in South Korea ^10^. Data regarding the pathogenicity to animals and zoonotic potential of this bacterium are limited. So far, only asymptomatic shedding of *C. gallinacea* has been reported in field studies conducted in birds. However, experimentally infected broilers showed reduced body-weight gains (BWG); this was the sole symptom and they did not exhibit any clinical signs of disease ^5^. Mortality in embryonated chicken eggs after inoculation with different *C. gallinacea* strains has also been described ^5,11^. Laroucau et al*.* ^12^ reported an outbreak of atypical pneumonia in slaughterhouse workers that were exposed to *C. gallinacea* infected poultry, thereby indicating that zoonotic transmission of *C. gallinacea* is plausible. The available studies characterize the strains of this species as genetically diverse ^13^; different strains were also recorded in Poland. In this study, strain 15-56/1 isolated from a Polish chicken flock was used for experimental inoculation of chicken broilers in order to evaluate the course of infection and its impact on production parameters.

## Materials and methods

### Ethics declaration

The study was conducted in accordance with the Act on the Protection of Animals Used for Scientific or Educational Purposes of 15 January, 2015 published in the Journal of Laws of 2015 as item 266 and the European regulations on the protection of animals used for scientific purposes (Directive 2010/63/EU). The animal experiment was carried out according to the requirements and with the approval of the Local Ethics Committee for Animal Testing at the University of Life Sciences in Lublin (decision no. 64/2019 of 18 September 2019). The experiment complied with the “Animal Research: Reporting of In Vivo Experiments (ARRIVE)” guidelines.

### Strain cultivation and inoculum preparation

*Chlamydia gallinacea* strain 15-56/1 was isolated from a cloacal swab taken from *Gallus gallus* based on a procedure published previously ^6,14^. The strain was propagated in a buffalo green monkey cell culture with UltraMDCK serum-free medium (Lonza, Germany) in T75 flasks. The medium was renewed after 18 h. The cultures were incubated at 37 °C with 5% CO_2_ in a fully humidified cabinet for 72 h. Density gradient centrifugation was applied to purify live elementary bodies ^15^. The pellet was resuspended in sucrose phosphate glutamate buffer. Subsequently, 200 µl of inoculum was used for DNA extraction with a DNeasy Blood & Tissue Kit (Qiagen, Germany) following the manufacturer’s instructions. Genomic DNA was checked quantitatively by means of a Qubit 3.0 fluorometer (Thermo Fisher Scientific, USA) to determine the number of genome copies. Molecular characterization of this strain was previously performed by Szymańska-Czerwińska et al*.* ^6^. Analyses revealed that it is genetically different from previously known European and Chinese strains, including the type strain 08-1274/3. The genome sequence of the isolate has not yet been published, but whole genome sequence (WGS) analyses are in progress.

### Chicken broilers and infection

A total of 30 1-day-old broiler chicks purchased from a commercial hatchery were used in this study. The experiment was conducted in the biosafety level 3+ animal facility (BSL3+) at the National Veterinary Research Institute, Poland and lasted seven weeks. Chickens were randomly divided into a control (n = 15) and an experimental group (n = 15) and housed in separate rooms in metal grid cages. They were reared for seven days for acclimatization. Water and feed were provided ad libitum during the whole experiment period. Animals were fed a commercial broiler starter diet to 21 days, a grower diet to 35 days and a finisher diet to 42 days of age. The feed did not contain coccidiostats. The temperature was manually controlled and gradually reduced from 32 to 33 °C in the first week to 20–21 °C at the end of the experiment. Chickens were first maintained on a 24-h light cycle, and then the light program was progressively changed to 18 h of light and 6 h of darkness. The number of air exchanges was 12 per hour and humidity was maintained between 55 and 70%. Prior to experimental inoculation, oropharyngeal and cloacal swabs were collected for real-time PCR analyses in order to exclude the possibility of *Chlamydiaceae* infection.

After one week of acclimatization, chickens from the experimental group were inoculated intranasally with 20 µl of 10^7^ genomes of *C. gallinacea* strain 15-56/1 and all birds in the control group received 20 µl of sterile PBS intranasally (applied dropwise using a pipette). At 2, 5, 7, 14, 21, 28, and 35 dpi oropharyngeal and cloacal swabs were collected from all chickens using commercial swabs with universal transport medium (Copan, Italy). It was not necessary to exclude or prematurely euthanize any animal out of concern for its welfare.

### Assessment of health and production parameters

Animals were monitored daily for the presence of any clinical signs of disease. Birds were individually weighed once a week, and starting from seven days of age BWG was calculated weekly using the difference between final and initial body weight. The feed consumed in each pen was recorded when the birds were weighed. Average weekly feed intake per bird was calculated as the feed consumed per week in the group divided by the number of birds in the group (n = 15). The feed conversion ratio (FCR = weekly feed intake divided by weekly weight gain) was determined for both groups for the same interval.

### Euthanasia and sampling

At 35 dpi the animals were humanely euthanized by isoflurane inhalation followed by immediate decapitation as previously approved by the Local Ethical Committee. Then the heart, trachea, lungs, liver, spleen, kidneys, stomach (proventriculus and gizzard), jejunum, caecum, rectum, testicles or ovaries and cloaca of all individuals were collected during the necropsy. Samples were subjected to further analysis and stored at − 20 °C before isolation of nucleic acids.

### DNA isolation and real-time PCR

Extraction of DNA from swabs and tissue specimens was performed using a QIAamp DNA Mini Kit and DNeasy Blood & Tissue Kit (Qiagen, Germany) according to the manufacturer’s instructions, but with one modification. DNA extracts were eluted in 100 µl of elution buffer instead of 200 µl. DNA extracts were stored at − 20 °C before analysis.

A real-time PCR was performed using a 7500 Fast Real-Time PCR system v2.3 (Applied Biosystems, USA) to detect a *Chlamydiaceae*-specific 23S rRNA gene fragment ^16^ in swabs collected from all birds prior to experimental inoculation. Identification of *C. gallinacea* in swabs and tissue samples was conducted by the amplification of an *eno*A gene fragment as described by Laroucau et al*.* ^17^. A panel of required positive and negative controls was used in each run, including TaqMan Exogenous Internal Positive Control (Thermo Fisher Scientific, USA) as a commercial internal amplification control to monitor PCR inhibitors. All samples with a cycle-threshold (Ct) value above 36 were considered negative. The cut-off value was selected based on the limit of detection determined in the validation process. These real-time PCR procedures were validated under laboratory conditions according to the Manual of Diagnostic Tests and Vaccines for Terrestrial Animals ^18^ and accredited by the Polish Centre for Accreditation.

### Statistical analyses

All statistical data analyses were conducted using Statistica software, version 10.0 (StatSoft, USA). The control of the normality of distributions was performed using the Shapiro–Wilk test, while the homogeneity of variance was tested with the Levene and Brown–Forsythe tests. The values obtained for the experimental and control group were analyzed using Student’s *t*-test. One exception was made for comparison of the FCR values: data from the experimental group collected in the fourth week post inoculation (wpi) were not normally distributed, and in this case, a Mann–Whitney test was applied.

## Results

No behavioral changes or clinical manifestations of infection were observed in any birds from either the control or experimental group throughout the whole study. The internal body temperature of all individuals did not exceed normal ranges, the birds were vigorous and they eagerly took feed.

The presence of *C. gallinacea* DNA was detected in oropharyngeal and cloacal swabs taken from animals in the experimental group from the fifth until the 35th dpi (Table 1). At five dpi 4/15 cloacal and 6/15 oropharyngeal swabs were positive and two days later the number of positive swabs had increased to 7/15 and 10/15, respectively. From the 14th dpi until the end of the experiment, all collected swabs were *C. gallinacea*-positive. Lower Ct values were noted for cloacal than oropharyngeal specimens.

**Table 1:** Results of testing of oropharyngeal and cloacal swabs for the presence of *C. gallinacea* DNA.

Days post inoculation		0 dpi		2 dpi		5 dpi		7 dpi		14 dpi		21 dpi		28 dpi		35 dpi	
No.	Bird ID	Type of swab															
		Oropharyngeal	Cloacal	Oropharyngeal	Cloacal	Oropharyngeal	Cloacal	**Oropharyngeal**	Cloacal	Oropharyngeal	Cloacal	Oropharyngeal	Cloacal	Oropharyngeal	Cloacal	Oropharyngeal	Cloacal
1	628	**−**	**−**	**−**	**−**	**−**	**−**	**+**	**+**	**+**	**+**	**+**	**+**	**+**	**+**	**+**	**+**
2	629	**−**	**−**	**−**	**−**	**+**	**+**	**+**	**+**	**+**	**+**	**+**	**+**	**+**	**+**	**+**	**+**
3	630	**−**	**−**	**−**	**−**	**+**	**+**	**+**	**+**	**+**	**+**	**+**	**+**	**+**	**+**	**+**	**+**
4	631	**−**	**−**	**−**	**−**	**−**	**−**	**−**	**−**	**+**	**+**	**+**	**+**	**+**	**+**	**+**	**+**
5	632	**−**	**−**	**−**	**−**	**−**	**−**	**+**	**+**	**+**	**+**	**+**	**+**	**+**	**+**	**+**	**+**
6	633	**−**	**−**	**−**	**−**	**+**	**+**	**+**	**+**	**+**	**+**	**+**	**+**	**+**	**+**	**+**	**+**
7	634	**−**	**−**	**−**	**−**	**−**	**−**	**−**	**−**	**+**	**+**	**+**	**+**	**+**	**+**	**+**	**+**
8	635	**−**	**−**	**−**	**−**	**−**	**−**	**−**	**−**	**+**	**+**	**+**	**+**	**+**	**+**	**+**	**+**
9	636	**−**	**−**	**−**	**−**	**+**	**−**	**+**	**+**	**+**	**+**	**+**	**+**	**+**	**+**	**+**	**+**
10	637	**−**	**−**	**−**	**−**	**−**	**−**	**−**	**−**	**+**	**+**	**+**	**+**	**+**	**+**	**+**	**+**
11	638	**−**	**−**	**−**	**−**	**+**	**−**	**+**	**−**	**+**	**+**	**+**	**+**	**+**	**+**	**+**	**+**
12	639	**−**	**−**	**−**	**−**	**−**	**−**	**−**	**−**	**+**	**+**	**+**	**+**	**+**	**+**	**+**	**+**
13	640	**−**	**−**	**−**	**−**	**+**	**+**	**+**	**+**	**+**	**+**	**+**	**+**	**+**	**+**	**+**	**+**
14	641	**−**	**−**	**−**	**−**	**−**	**−**	**+**	**−**	**+**	**+**	**+**	**+**	**+**	**+**	**+**	**+**
15	642	**−**	**−**	**−**	**−**	**−**	**−**	**+**	**−**	**+**	**+**	**+**	**+**	**+**	**+**	**+**	**+**
Total		0	0	0	0	6	4	10	7	15	15	15	15	15	15	15	15

No macroscopic pathological lesions were observed in necropsies of inoculated animals. Detection of chlamydiae in collected tissues using microscopy-based methods was not performed. Testing of internal organ homogenates confirmed the presence of pathogen DNA in the trachea, lungs, kidney, stomach, jejunum, cecum, rectum, testicles or ovaries and cloaca of *C. gallinacea*-inoculated birds (Table 2). All cecum and colon specimens and all but one cloaca and trachea samples were positive; however, *C. gallinacea* DNA was not found in the heart, liver or spleen. The lowest mean Ct value was noted in the cecum (25.66) followed the colon (27.6) and cloaca samples (28.2). All cloacal and oropharyngeal swabs as well as the internal organs collected from the control group were negative for the presence of *C. gallinacea* DNA.

**Table 2:** Ct values obtained for the detection of *C. gallinacea* DNA in organs of inoculated broilers.

Bird ID	Organ											
	Stomach	Jejunum	Cecum	Colon	Cloaca	Kidney	Testicle or ovary	Trachea	Lungs	Heart	Spleen	Liver
628	–	–	30.02	33.42	24.91	–	–	34.89	–	–	–	–
629	34.21	29.75	22.34	21.72	24.68	–	–	30.44	34.02	–	–	–
630	–	–	25.74	24.76	30.89	–	–	30.67	35.76	–	–	–
631	33.52	32.93	27.86	25.19	22.51	36.00	–	21.94	34.02	–	–	–
632	–	35.53	24.36	27.38	26.79	–	–	35.94	–	–	–	–
633	32.92	–	24.01	29.65	29.72	35.16	33.32	28.25	–	–	–	–
634	33.17	35.44	21.83	27.26	30.95	–	–	32.68	–	–	–	–
635	–	–	28.98	30.72	30.55	–	–	30.74	–	–	–	–
636	35.70	35.61	27.96	29.61	28.57	–	–	34.85	35.92	–	–	–
637	–	–	24.25	25.57	29.36	–	–	31.22	31.92	–	–	–
638	33.71	32.77	25.35	28.29	27.71	33.82	–	–	–	–	–	–
639	–	35.98	24.21	24.16	27.69	–	–	27.86	–	–	–	–
640	–	–	28.64	26.79	26.92	–	–	34.38	–	–	–	–
641	35.32	–	22.81	29.56	–	–	–	30.07	–	–	–	–
642	–	35.91	26.60	29.92	33.61	–	–	30.89	–	–	–	–
Total (mean Ct value)	7/15 (34.08)	8/15 (34.24)	15/15 (25.66)	15/15 (27.6)	14/15 (28.2)	3/15 (34.99)	1/15 (33.32)	14/15 (31.06)	5/15 (34.33)	0	0	0

**Figure 1:**
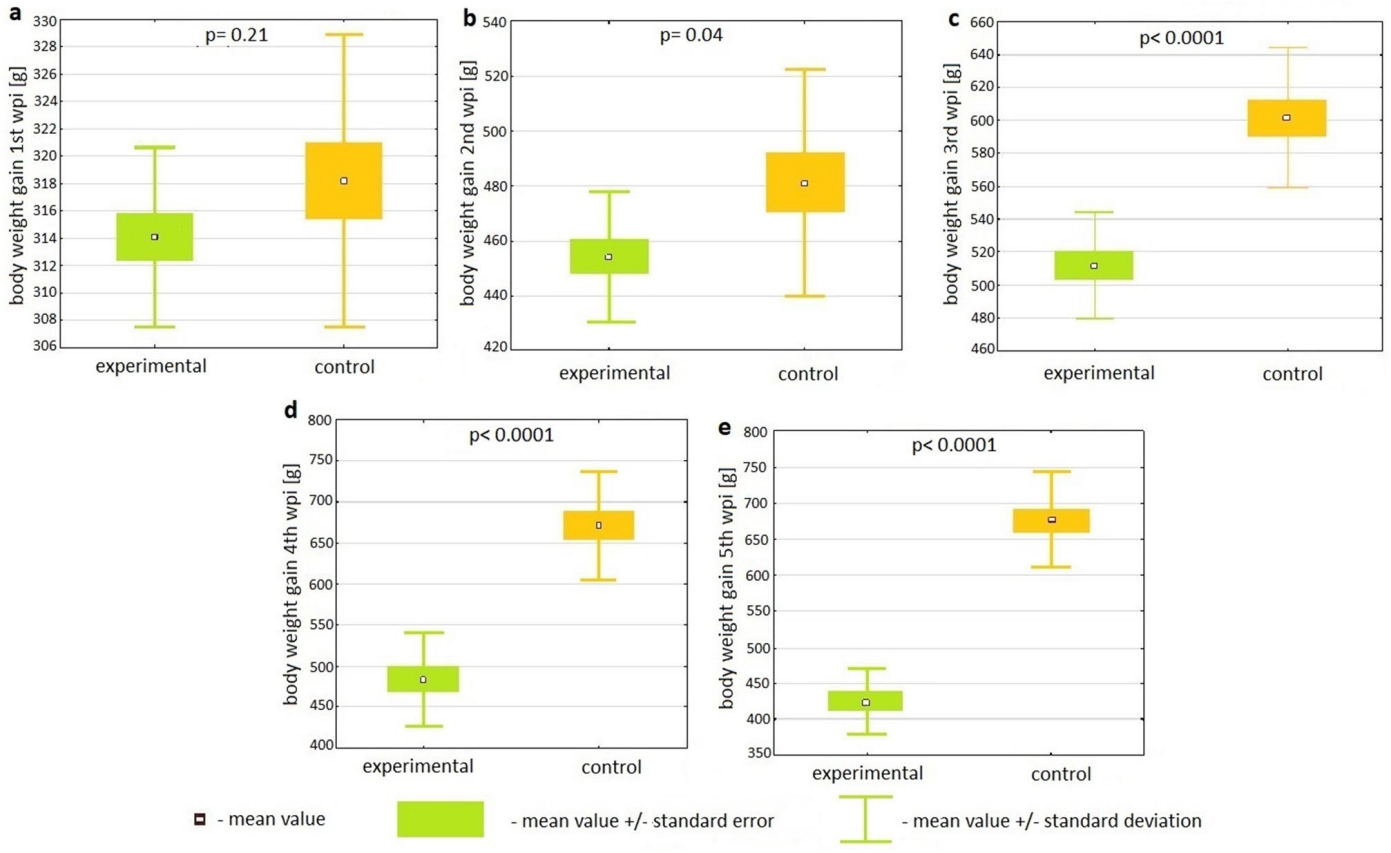
Box plots depicting the differences in weekly body weight gain between the control and experimental groups in the first (**a**), second (**b**), third (**c**), fourth (**d**) and fifth (**e**) weeks post inoculation with *C. gallinacea.* The difference between groups is statistically significant if p < 0.05; *wpi* week post inoculation.

The weight of the broiler chicks prior to inoculation (seventh day of life) did not differ significantly among the groups and amounted to averages of 199.27 g and 197.13 g for the control and experimental groups. Statistical analyses showed that broilers in the experimental group exhibited lower BWG than animals in the control group (Fig. 1), which started at 4.87% in the second wpi, changed to 7.52% in the third and 9.36% in the fourth, and ended at 8.21% in the fifth week post inoculation. Differences in weekly BWG were statistically significant from the second wpi until the end of experiment (p = 0.04 for the second week and p < 0.0001 for each of the next three). Detailed results are presented in Fig. 1. Statistical analyses of the differences in weekly BWG between the control and experimental groups using Student’s *t*-test are shown in Supplementary Table S1.

**Figure 2:**
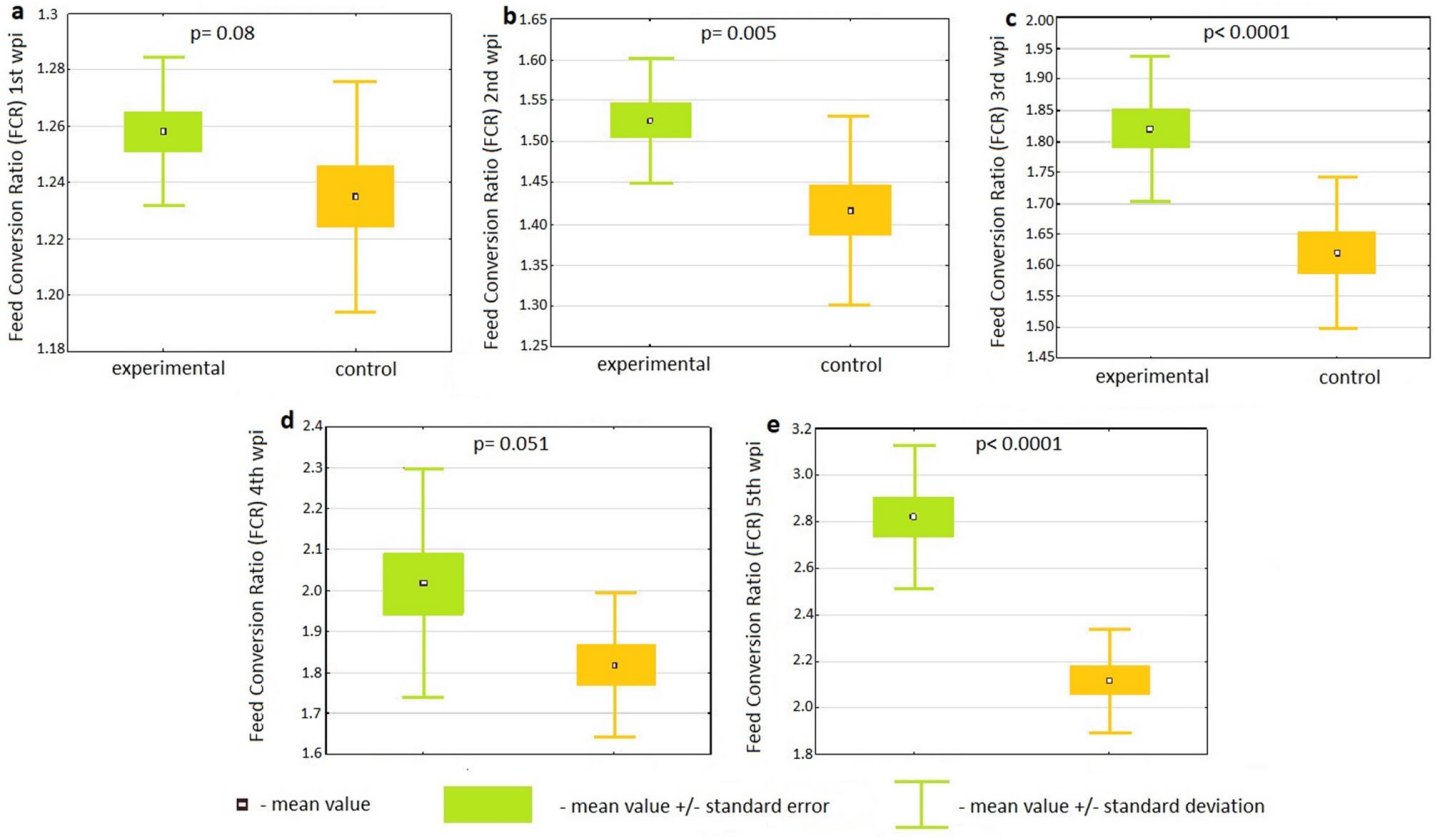
Box plots illustrating the differences in feed conversion ratios between the control and experimental groups in the first (**a**), second (**b**), third (**c**), fourth (**d**) and fifth (**e**) weeks post inoculation with *C. gallinacea*. The difference between groups is statistically significant if p < 0.05; *wpi* week post inoculation.

Calculated FCRs were also worse (higher) in the experimental group (Fig. 2). Except for the first wpi (p = 0.08), the FCR differences were statistically significant (p = 0.005 for the second and p < 0.0001 for the third and fifth). Supplementary Table S2 presents detailed results of the statistical analyses of differences in FCRs between the control and experimental groups using Student’s *t*-test. The results of the Mann–Whitney U test for the fourth wpi are set out in Supplementary Table S1, and were at the borderline significance level (p = 0.051).

## Discussion

*Chlamydia gallinacea* is a newly identified pathogen and still little is known about its pathogenicity and inter-strain genetic diversity. Research data from the USA, Mexico, China and some European countries indicate that this bacterium is widespread in domestic poultry ^5,7,19–21^. In Poland, which is the European Union leader in the production and export of poultry, *C. gallinacea* has been the most prevalent *Chlamydia* species, detected in 65% of positive poultry flocks ^6^.

This is the first study that describes the course of infection after experimental inoculation of chicken broilers with a Polish *C. gallinacea* strain and its impact on production parameters, i.e. BWG and FCR.

In this research, broiler chickens experimentally infected with the Polish *C. gallinacea* strain (15-56/1) showed lower BWG and higher FCR in comparison to birds in the control group. Significantly lower gains were observed from the second week post inoculation, which is congruent with data presented by Guo et al*.* ^5^. The percentage difference in BWG increased progressively from the second wpi until the fourth wpi. In the fifth wpi, BWG in the experimental group was significantly lower than in the control group, but the percentage difference was smaller than in the previous week. Similarly to previous reports, bacteria were detected in both cloacal and oropharyngeal swabs, and infection in all birds remained subclinical ^22^. The presence of *C. gallinacea* DNA was detected in swabs from 5 dpi. In tissue, the pathogen was present in all but one trachea sample but only in 5/15 (33%) lung specimens, which may suggest that *C. gallinacea* prefers the trachea over the lung as a niche for the replication. Further studies, including histopathological analyses of air sacs, need to be performed to evaluate if this phenomenon occurs.

Despite the asymptomatic course, infection in broilers resulted in significantly lower final body weight. Additionally, an animal’s efficiency in converting feed into increased body mass (the FCR) was substantially worse in the experimental group. These factors may generate serious economic losses and increase the cost of broiler chicken farming.

In this study, the highest bacterial DNA load was detected in the cecum, colon and cloaca. These findings are largely congruent with the results of research conducted by Heijne et al*.* ^23^. The bacterial distribution confirmed *C. gallinacea* infection mainly resides in distal parts of the gut, and immunohistochemistry of tissue samples revealed that the bacteria infected the epithelium of the jejunum, ileum and caecum but not the colon. No pathological lesions in the internal organs of infected chickens were observed in the studies. However, further investigations are essential for evaluation of whether the presence of the pathogen in the intestines may impair the function of intestinal cells.

Guo et al*.* ^5^ reported mortality of chicken embryos after yolk sac inoculation with *C. gallinacea*. Similarly, Heijne et al*.* ^11^ observed *C. gallinacea*-induced mortality in experimentally infected embryonated chicken eggs, which was potentially strain dependent but lower than *C. psittaci*-induced mortality. According to You et al*.* ^22^, vertical transmission of the bacteria can occur via eggshell penetration. The pathogen can penetrate from the eggshell to albumen, yolk and growing embryos, even despite the protection of the egg membrane. As a consequence, asymptomatic infection of breeding flocks may lead to spreading of the disease via contaminated or infected eggs. As further elucidation of the bacterium’s routes of spread, efficient transmission of *C. gallinacea* via the fecal–oral route was observed in the same study; however it was not noted via aerosol. It is unknown if subclinical *C. gallinacea* infection may facilitate bacterial/viral co-infections and whether co-infections might exacerbate the disease outcome.

So far, only asymptomatic shedding of *C. gallinacea* has been reported, although molecular genotyping studies revealed genetic diversity among *C. gallinacea* strains ^13^ and detailed comparative genomic analysis showed the presence of genes that are considered to be related to chlamydial virulence ^11,13,24^. More genotyping and experimental studies need to be conducted to evaluate if genetic diversity results in differences in pathogenicity and virulence of the various strains.

In summary, experimental inoculation of chicken broilers with *C. gallinacea* strain 15-56/1 resulted in lower body weight gain, worse FCRs and asymptomatic shedding of bacteria by infected birds in oropharyngeal and cloacal swabs. These results are consistent with previous studies and indicate that *C. gallinacea* possibly causes persistent, subclinical infection leading to a negative effect on production parameters. Further studies, including genomic analyses, will be conducted to fully characterize the strains isolated from birds in Poland.

## Supplementary Information

Supplementary Information.

## Data Availability

Data generated and analysed during this study are presented in Tables and Figures. The raw data are available from the corresponding author upon reasonable request.

## Abbreviations

BSL3+: Biosafety level 3+
BWG: Body weight gain
Ct: Cycle threshold
dpi: Days post-inoculation
FCR: Food conversion ratio
PBS: Phosphate buffered saline
wpi: Week post-inoculation
