# Supplementary material for: Experimental inoculation of chicken broilers with C. gallinacea strain 15-56/1
Source: Sci Rep. 2021 Dec 13;11:23856. doi: 10.1038/s41598-021-03223-w (PMC8668880; doi:10.1038/s41598-021-03223-w)
Supplement: Supplementary file 1 — Supplementary Information. [file 41598_2021_3223_MOESM1_ESM.pdf]

Experimental inoculation of chicken broilers with

C. gallinacea strain 15-56/1

Monika Szymańska-Czerwińska, Agnieszka Jodełko, Kinga Zaręba-Marchewka, Krzysztof Niemczuk

Supplementary Information

Supplementary Table S1 Results of statistical analyses of differences in weekly body weight gain (BWG) between the control and experimental groups using Student's *t*-test.

BWG after inoculation	Mean BWG in experimental group	Mean BWG in control group	t	df	p-value*	Valid N control group	Valid N experimental group	SD for experimental group	SD for control group
1st week	314.0667	318.2	−1.2755	28	0.212621	15	15	2.669847	10.70514
2nd week	454.2	481.0667	−2.1826	28	0.037610	15	15	23.86629	41.26996
3rd week	511.6	601.8	−6.5471	28	0.000000	15	15	32.30170	42.47049
4th week	482.8667	671.4	−8.3114	28	0.000000	15	15	57.36583	66.53871
5th week	424.6	677.533	−12.2360	28	0.000000	15	15	45.80830	65.65915

t–computed test statistic; *df*– degrees of freedom; *p-value of <0.05 was considered to be statistically significant

Supplementary Table S2 Results of statistical analyses of differences in FCRs between the control and experimental groups using Student's *t*-test.

FCR after inoculation	Mean FRC in experimental group	Mean FCR in control group	t	df	p-value*	Valid N control group	Valid N experimental group	SD for experimental group	SD for control group
1st week	1.258207	1.234899	1.845481	28	0.075568	15	15	0.026324	0.041227
2nd week	1.525984	1.416872	3.061966	28	0.004816	15	15	0.076604	0.114801
3rd week	1.820972	1.621201	4.624497	28	0.000077	15	15	0.115659	0.120890
4th week	2.019043	1.816392	2.365965	28	0,025140	15	15	0.282193	0.174391
5th week	2.821262	2.118691	7.188275	28	0.000000	15	15	0.306382	0.222312

t–computed test statistic; *df*– degrees of freedom; *p-value of <0.05 was considered to be statistically significant

Supplementary Table S3 Results of statistical analyses of differences between the control and experimental groups in FCRs in the 4th wpi (Mann–Whitney U test with continuity correction).

FCR after inoculation	Sum of ranks for experimental group	Sum of ranks for control group	U	Z	p-value*	Z-corrected	p-value	Valid N experimental group	Valid N control group	Exact Sig. [2*(one-sided Sig.)]
4th week	280.0	185.0	65.0	1.949469	0.05124	1.949469	0.05124	15	15	0.050222

sig – significance; *p-value of <0.05 was considered to be statistically significant
